# Interaction between warfarin and short-term intravenous amiodarone in intensive care unit patients after cardiac surgery

**DOI:** 10.1186/s40780-018-0110-6

**Published:** 2018-05-30

**Authors:** Tomoki Takase, Hiroaki Ikesue, Makiko Tohi, Hiroshi Ueta, Hiroyuki Mima, Tadaaki Koyama, Tohru Hashida

**Affiliations:** 1 Department of Pharmacy, Kobe City Medical Center General Hospital, 2-2-1, Minatojima Minamimachi, Chuo-ku, Kobe, Hyogo 650-0047 Japan; 2 Department of Anesthesiology and Critical Care, Kobe City Medical Center General Hospital, 2-2-1, Minatojima Minamimachi, Chuo-ku, Kobe, Hyogo 650-0047 Japan; 3 Department of Cardiovascular Surgery, Kobe City Medical Center General Hospital, 2-2-1, Minatojima Minamimachi, Chuo-ku, Kobe, Hyogo 650-0047 Japan

**Keywords:** Warfarin, Amiodarone, Interaction, Intravenous, Prothrombin time, Cardiac surgery, Intensive care unit, Short-term

## Abstract

**Background:**

Amiodarone and warfarin are sometimes administered immediately after cardiac surgery. Although the interaction between long-term oral amiodarone and warfarin has been reported, the interaction between warfarin and short-term intravenous amiodarone has not been reported. In this study, we investigated the effect of short-term intravenous amiodarone on the anticoagulant effect of warfarin in patients who underwent cardiac surgery.

**Methods:**

We retrospectively reviewed the medical records of 11 patients who received oral warfarin before and after cardiac surgery, and loading doses of 125–150 mg or a 750 mg continuous infusion of amiodarone, or both in the intensive care unit (ICU) within 5 days after the surgery between July 2011 and January 2017. The prothrombin time-international normalized ratio (PT-INR)/daily warfarin dose (PT-INR/dose) was used as an indicator of anticoagulant effect. The values before surgery were considered as the baseline.

**Results:**

The PT-INR and PT-INR/dose values were elevated in 7 and 10 patients, respectively, after amiodarone administration. The mean PT-INR values were not significantly different before and after amiodarone administration (2.13 ± 0.58 vs 2.29 ± 0.50, respectively, *p* = 0.643). In contrast, the mean PT-INR/dose values were significantly elevated after the administration of amiodarone (0.93 ± 0.46 vs 1.54 ± 0.63, respectively, *p* = 0.002).

**Conclusions:**

Short-term intravenous amiodarone enhanced the anticoagulant effect of warfarin in patients admitted to the ICU after cardiac surgery. We suggest that the dose of warfarin should be carefully adjusted for a few days after cardiac surgery if intravenous amiodarone is coadministered.

## Background

Warfarin is an essential drug for the prevention of thrombosis after valvular surgery, which has a narrow therapeutic range. Although there are general dosing guidelines, the dosing of warfarin is complicated by relatively high incidences of drug-drug interactions and inter-patient variability [1–3]. Controlling the dosage of warfarin in patients admitted to the intensive care unit (ICU) immediately after cardiac surgery can be especially complicated by multiple factors including unstable patient condition and concomitant use of various medication [4, 5]. Moreover, the altered pharmacokinetic parameters reported in these critical patients compared with those who are non-critically ill contributes to the challenge of controlling the warfarin dose [6].

Postoperative atrial fibrillation after cardiac surgeries has been reported in approximately 30% of cases, and short-term intravenous amiodarone is sometimes necessary for treatment [7]. Amiodarone is known to induce drug-drug interactions with warfarin that could lead to excessive anticoagulation and bleeding risk [8–12]. Similar to warfarin, amiodarone is metabolized in the liver by the drug-metabolizing enzyme cytochrome P450 (CYP), which it inhibits. The mechanism of the drug-drug interaction between warfarin and amiodarone is due to the inhibition of CYP. Amiodarone and its active metabolite “desethylamiodarone” inhibit CYP2C9, which increases the anticoagulation effect of warfarin [8].

Amiodarone has unique pharmacokinetic properties, and following oral administration, it is approximately 40% bioavailable and 96% plasma protein bound. The mean half-life of amiodarone is 40–55 days [13–15]. Therefore, it may require 130–535 days (five half-lives) for amiodarone to reach steady-state levels. To date, various studies have reported drug-drug interactions between warfarin and long-term oral amiodarone administration [8–12]. However, little is known about the interactions between warfarin and intravenous amiodarone [9]. To our knowledge, there have not been any previously reported on the drug-drug interaction between warfarin and short-term intravenous amiodarone in an ICU setting. Thus, we investigated the effect of short-term intravenous amiodarone on the anticoagulant effect of warfarin in patients who underwent cardiac surgery.

## Methods

This retrospective study protocol was approved by the institutional review board of the Kobe City Medical Center General Hospital, Japan (Approval No. zn170806). Patient characteristics including age, sex, indications for receiving warfarin and amiodarone, the dose of warfarin and amiodarone, prothrombin time-international normalized ratio (PT-INR), and underlying organic heart diseases were reviewed using the electronic medical record system. Patients who received oral warfarin before and after cardiac surgery, as well as loading doses of 125 to 150 mg or subsequent continuous infusion of 750 mg amiodarone or both in the ICU within 5 days after surgery between July 1, 2011, and January 31, 2017 were selected as the amiodarone group (*n* = 11). Consecutive 15 patients who received oral warfarin before and after cardiac surgery without amiodarone between May 1, 2016, and January 31, 2017 were selected as the control group (*n* = 15).

Patients were excluded from both study groups if they received oral amiodarone or other drugs (fluconazole, miconazole, bucolome, benzbromarone, rifampicin) known to markedly alter the effect of warfarin during the study period [16]. In the amiodarone group, among 307 patients admitted to the ICU and received intravenous amiodarone, 25 patients have received warfarin before cardiac surgery. Fourteen patients were excluded because they received oral amiodarone (*n* = 8), or bucolome (*n* = 1) or other medication (*n* = 5). Residual 11 patients met the inclusion criteria and were followed up for 15 days after the initial dose of intravenous amiodarone.

In the both study groups, all patients received cefazolin for 3 days followed by surgery for the prevention of surgical site infection. The PT-INR values were frequently monitored during the study period, especially after the patients were admitted to the ICU. The daily doses of warfarin were adjusted as necessary to maintain a therapeutic PT-INR of 1.5–2.0, which is suitable for Japanese patients [17, 18]. The PT-INR/daily dose of warfarin (PT-INR/dose) was used as an indicator of the anticoagulant effect [19]. In this study, PT-INR/dose was calculated as follows:

$$ \mathrm{PT}\hbox{-} \mathrm{INR}/\mathrm{dose}=\mathrm{PT}\hbox{-} \mathrm{INR}/\mathrm{dose}\ \mathrm{of}\ \mathrm{warfarin}\ \mathrm{of}\ \mathrm{the}\ \mathrm{previous}\ \mathrm{day}. $$

Before the surgery, the daily doses of warfarin, PT-INR value, and PT-INR/dose ratio were stable in the study subjects and were considered as the baseline values.

### Statistical analysis

All data are expressed as the mean ± standard deviation. Statistical analyses were performed using the JMP Pro 12.2.0 (SAS Institute Inc., Cary, NC, USA). To compare categorical data, the Chi-square test was used. For continuous data, values were presented as mean ± standard deviation (SD). Student’s *t*-test was used to compare groups. The changes in PT-INR and PT-INR/dose were analyzed using a paired *t*-test. All *p*-values < 0.05 were considered statistically significant.

## Results

The patient characteristics are shown in Table 1. Valvular heart disease was the most common cardiovascular comorbidity. In the baseline value, dosage of warfarin and PT-INR value were not significantly different in the both groups. Except for cefazoline, there was no change in combination of drugs known to influence pharmacokinetics and pharmacodynamics of warfarin.

**Table 1:** Patient characteristics

	Amiodarone (*n* = 11)	Control (*n* = 15)	*p*-values
Age, years (mean ± SD)	67.2 ± 7.5	71.7 ± 6.5	0.100
Men/Women, n	4/7	6/9	0.851
Cardiovascular comorbidity, n			–
Valvular heart disease	8	13	
Ischemic heart disease	1	0	
Thoracic aortic aneurysm	1	0	
Aortic dissection	0	1	
Aortic dissection + Ischemic heart disease	1	0	
Valvular heart disease + Ischemic heart disease	0	1	
Warfarin indication (preoperative), n			–
Atrial fibrillation	4	11	
Post prosthetic valve replacement	4	4	
Low LVEF	3	0	
Dosage of warfarin (baseline), mg/day (mean ± SD)	2.64 ± 0.89	3.07 ± 1.22	0.370
PT-INR value (baseline) (mean ± SD)	2.13 ± 0.58	2.27 ± 0.58	0.551

*LVEF* left ventricular ejection fraction

### Changes in mean PT-INR values and PT-INR/dose

**Fig. 1:**
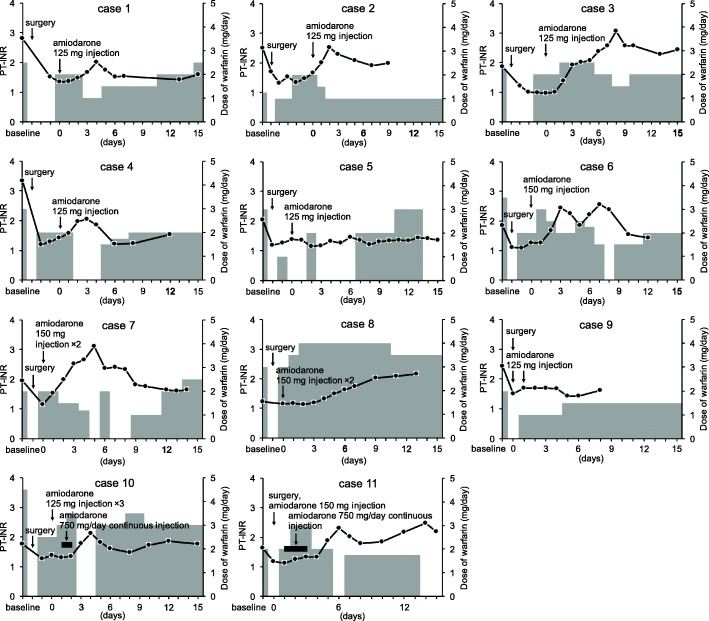
Change in values of prothrombin time-international normalized ratio (PT-INR, solid line, left line) and warfarin dosage (gray area, right axis) in the amiodarone group. X-axis shows days after administration of amiodarone

Changes in the PT-INR values and dosages of warfarin in all patients before and after the amiodarone injection are shown in Fig. 1. Compared with baseline values, those of the PT-INR were increased in seven patients (63.6%) (cases 2, 3, 6, 7, 8, 10 and 11) after the administration of amiodarone. Except for one patient (case 8), the time points which the daily warfarin doses were reduced after amiodarone injection were observed.

**Fig. 2:**
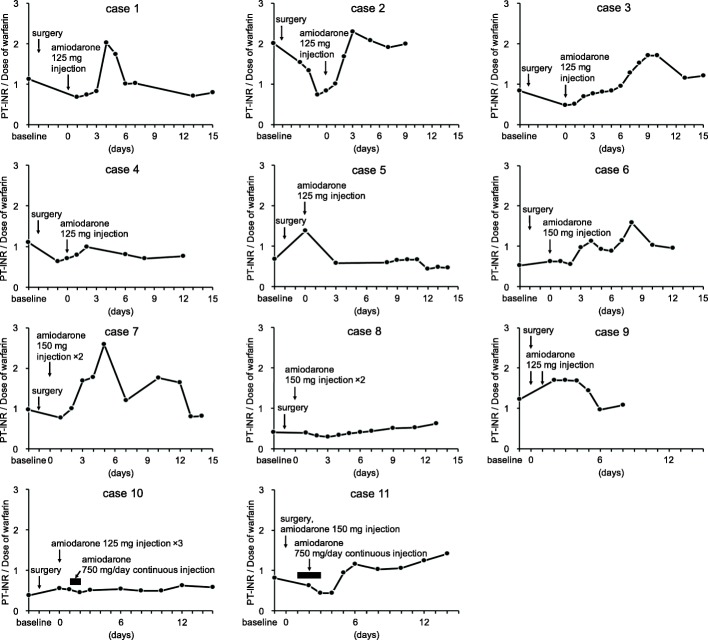
Change in prothrombin time-international normalized ratio/daily warfarin dose (PT-INR/dose) values (solid line) in the amiodarone group. X-axis shows days after administration of amiodarone

The change in PT-INR/dose values of all patients is shown in Fig. 2. The PT-INR/dose ratio increased in 10 patients (90.9%) (cases 1, 2, 3, 5, 6, 7, 8, 9, 10 and 11) after administration of amiodarone. Although the median time to reach the peak value of PT-INR/dose was 5 days after amiodarone injection, the values were not consistent in each case. Summarized data in the amiodarone group are shown in Table 2.

**Table 2:** Time to reach the peak values and change in values after amiodarone injection in the amiodarone group (*n* = 11)

	Median (range)
Time to reach the peak values (days)	
PT-INR	5 (2–14)
PT-INR / Dose of warfarin	5 (2–14)
Change in values from baseline to the maximal value	
PT-INR	0.36 (−1.61–1.19)
PT-INR / Dose of warfarin	0.60 (−0.23–1.62)

**Fig. 3:**
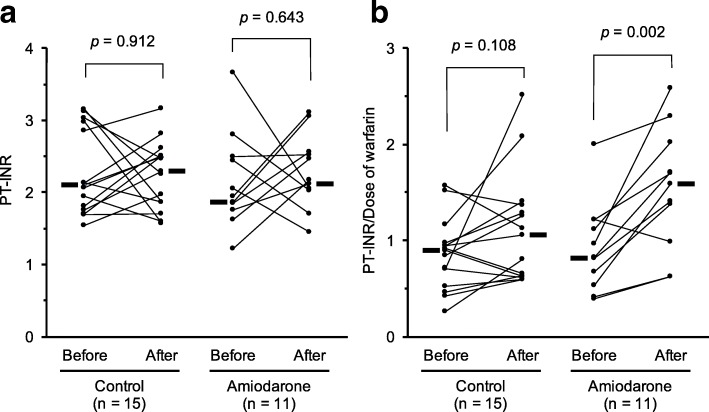
Changes in **a** prothrombin time-international normalized ratio (PT-INR) and **b** PT-INR/daily warfarin dose (PT-INR/dose) before and after surgery in the control (*n* = 15) and the amiodarone (*n* = 11) groups. The latter represents maximal value during study period. The bold lines represent the median values

The mean PT-INR values were not significantly different before and after surgery in the control group (2.27 ± 0.58 vs 2.25 ± 0.47, respectively, *p* = 0.912, Fig. 3a) and amiodarone group (2.13 ± 0.58 vs 2.29 ± 0.50, respectively, *p* = 0.643, Fig. 3a).

Although the mean PT-INR/dose values were not significantly elevated after surgery compared with the baseline value in the control group (0.86 ± 0.36 vs 1.11 ± 0.56, respectively, *p* = 0.108, Fig. 3b), those values were significantly elevated compared with the baseline value in the amiodarone group (0.92 ± 0.45 vs 1.54 ± 0.62 respectively, *p* = 0.002, Fig. 3b).

## Discussion

In this study, we investigated the effect of short-term intravenous amiodarone on the anticoagulant effect of warfarin in patients admitted to the ICU after cardiac surgery. The result showed that in most patients, the dosage of warfarin was reduced and the PT-INR, as well as the PT-INR/dose, were elevated after the administration of amiodarone compared with baseline values. The PT-INR values were carefully monitored, and warfarin dosages were frequently adjusted, and the maximal PT-INR values were not different from the baseline values both in the control and amiodarone groups. In contrast, the mean maximal PT-INR/dose values after intravenous amiodarone treatment were significantly elevated only in the amiodarone group compared to the baseline, and the timing of elevation was not consistent between cases.

Previous studies reported that co-administration of warfarin and amiodarone increased the bleeding risk by enhancing the anticoagulant effect of warfarin [9–12, 20, 21]. In out-patients who received both warfarin and amiodarone long-term, the drug-drug interaction induced by their co-administration was observed several weeks after co-treatment was started [9–12]. On the other hand, Edwin et al. [9] evaluated drug-drug interactions in patients who were coadministered amiodarone and warfarin and were admitted to the hospital [9]. They reported that the rate of PT-INR values > 2 observed 4 days after the initiation of warfarin treatment was higher in the group coadministered warfarin and amiodarone than in the group treated with warfarin alone [9]. Although they reported short-term amiodarone treatment, they only included patients who received both drugs for at least 4–5 days. In addition, the mean amiodarone dose in that study was approximately 1000 mg/day, which was higher than those in our study. We focused on the effect of amiodarone injections administered for 1–3 day only on the anticoagulant effect of warfarin in patients admitted to the ICU after cardiac surgery. The result showed that the anticoagulant effect was increased by amiodarone, even following this short-term treatment.

After cardiac surgery, some drugs added after surgery. However, we excluded patients who received medications which can strongly influence pharmacokinetics and pharmacodynamics of warfarin [16]. Cefazolin is known to influence the effect of warfarin, it was administered for all patients in the amiodarone and control group. Except for cefazoline, there was no change in combination of drugs known to influence pharmacokinetics and pharmacodynamics of warfarin. Therefore, we believe the increase in PT-INR/dose was affected by amiodarone. Warfarin is a racemic mixture, and the anticoagulant effect of (S)-warfarin is 5-folds stronger than that of (R)-warfarin is. The (S)- and (R)-warfarin enantiomers are metabolized by the drug-metabolizing CYP2C9 and CYP3A4 enzymes, respectively [4]. In this study, we considered that the inhibition of CYP2C9 by amiodarone and its active metabolite desethylamiodarone increased the anticoagulant effect of warfarin. Frequent dose adjustment of warfarin appeared to maintain the PT-INR values within the therapeutic range, and no significant difference occurred in the PT-INR values between baseline and after administration of amiodarone. In contrast, the PT-INR/dose values were elevated after administration of amiodarone in most patients. We considered that the administration of amiodarone could elevate the PT-INR/dose. In addition, the median time to achieve the peak value of the PT-INR/dose was 5 days after amiodarone injection. This time lag between the amiodarone injection and the peak time of the PT-INR/dose can be explained by the large volume of distribution and the long half-life of amiodarone [13, 14]. Although the PT-INR values were monitored daily, in patients admitted to the ICU after cardiac surgery, complex pharmacotherapy with multiple medications make it difficult to control the PT-INR values within an adequate range. In addition, the decrease in coagulation factors by surgical invasion with cardiopulmonary bypass and the dilution following the administration of crystalloids could also make it more difficult to control the anticoagulation status [5]. This study is useful because it partially explains the observed variation in the PT-INR.

This study has some limitations. First, it appears that not only amiodarone injection, but also cardiac surgery can affect the anticoagulant effect of warfarin. Previous studies have suggested that the PT-INR values in patients who underwent cardiac surgeries were increased after surgery compared with the values before surgery [22]. In our study, although the values of PT-INR/dose were not significantly different between before and after surgery without administration of amiodarone, the status after cardiac surgery individually can be different. In fact, the values of PT-INR/dose were not increased in some patients. We speculate that genetic polymorphisms of CYP2C9 and/or difference of volume of distribution in each individual may affect the degree of this dug-drug interaction.

Second, not all the PT-INR values were measured during the study period. During the 7.5-day mean ICU stay of these patients, they were closely monitored to minimize bleeding. However, after the patients were moved to general wards, the PT-INR was not monitored daily. Finally, the study retrospectively evaluated a small sample size. To overcome these limitations, further research is needed to compare the PT-INR values between patients receiving warfarin with or without short-term amiodarone treatment in larger sample sizes.

## Conclusions

To the best of our knowledge, this is the first study to show that short-term administration of intravenous amiodarone enhanced the anticoagulant effect of warfarin in patients admitted to the ICU after cardiac surgery. This study also demonstrated that PT-INR values can be elevated for several days after amiodarone injection. Therefore, the dose of warfarin needs to be carefully adjusted to minimize the risk of bleeding after cardiac surgery when intravenous amiodarone is coadministered.

## Abbreviations

AF: Atrial fibrillation
AFL: Atrial flutter
CYP: Cytochrome P450
ICU: Intensive care unit
LVEF: Left ventricular ejection fraction
PT-INR: Prothrombin time-international normalized ratio
PVC: Premature venticular contraction
VT: Ventricular tachycardia
